# Expression Regulation of Water Reabsorption Genes and Transcription Factors in the Kidneys of *Lepus yarkandensis*

**DOI:** 10.3389/fphys.2022.856427

**Published:** 2022-05-26

**Authors:** Shengjie Luo, Yongle Li, Shuwei Li, Renjun Jiang, Fang Deng, Guoquan Liu, Jianping Zhang

**Affiliations:** ^1^ College of Life Sciences and Technology, Tarim University, Alar, China; ^2^ Xinjiang Production and Construction Corps Key Laboratory of Protection and Utilization of Biological Resources, Tarim University, Alar, China; ^3^ Anhui Province Key Laboratory of Translational Cancer Research and Department of Biochemistry, College of Laboratory Medicine, Bengbu Medical College, Bengbu, China; ^4^ College of Animal Science and Veterinary Medicine, Huazhong Agricultural University, Wuhan, China

**Keywords:** *Lepus yarkandensis*, transcription factor, kidney, water reabsorption, environmental adaptation

## Abstract

*Lepus yarkandensis* is a desert-dwelling animal that has various adaptations to cope with drought. The kidney maintains water and acid-base balance mainly through the vasopressin-regulated water reabsorption pathway and proximal tubular bicarbonate reabsorption pathway. In this study, we compared the differentially expressed genes (DEGs) and transcription factors in the kidneys of *L. yarkandensis* and *Oryctolagus cuniculus* to explore the relationship between the DEGs in kidneys and the animals’ adaptations. Transcriptome sequencing data were used to predict the differentially-expressed water reabsorption genes and their transcription factors. Quantitative real-time PCR, immunohistochemistry, and western blotting were used to detect and verify the expression of DEGs in the kidney at mRNA and protein levels. Transcriptome analysis of the kidney of *L. yarkandensis* and *O. cuniculus* showed that 6,610 genes were up-regulated and 5,727 genes down-regulated in data shared by both species. According to the data, 232 transcription factors and their corresponding target genes were predicted, from which genes and transcription factors related to renal water reabsorption were screened. Quantitative RT-PCR results showed *AQP1*, *AQP2*, *ADCY3*, *HIF1A*, *CREB3*, and *NFATc1* had higher expression in the *L. yarkandensis* kidney; in comparison, *FXYD2* mRNA expression levels were lower. In western blotting, transcription factors *HIF1A*, *NFATc1*, *NF-κB1*, and critical genes *ADCY3*, *ATPA1*, and *SLC4A4*, were highly expressed in the kidneys of *L. yarkandensis*. Immunohistochemical results showed that the *ADCY3* protein was in the basolateral membrane of the collecting duct, the *ATP1A1* protein was in the basolateral membrane and medulla of proximal tubules, and the *SLC4A4* protein was in the basolateral membrane of proximal tubules. According to these results can be inferred that *HIF1A*, *NFATc1*, and *NF-κB1* play a certain role in regulating the expression of genes related to water reabsorption in the kidney of *L. yarkandensis*, thus improving the water reclamation efficiency of *L. yarkandensis*, so as to adapt to the arid desert environment.

## Introduction

*Lepus yarkandensis* (Yarkand hare), belonging to Leporidae and Lagomorpha, is endemic to deserts of the Tarim Basin, Xinjiang, China. The Tarim Basin has an arid continental climate, with warm, dry air in summer and cold air in winter, with a big difference in temperature between day and night. As such, *L. yarkandensis* must cope with extreme aridity, intense solar radiation, and have a high heat tolerance. Compared with *Oryctolagus cuniculus* and other hares, *L. yarkandensis* has many specific adaptations to arid environments. These adaptations have been explored using SNP analysis, population structure (Ababaikeri et al., 2021), mitochondrial genome sequencing, phylogenetic tree construction (Shan et al., 2021), as well as aquaporin (*AQP*) expression in the kidney, gastrointestinal tract, and lungs (Zhang et al., 2019a; Zhang et al., 2019b; Zhang et al., 2020).

For mammals, controlling osmotic pressure in the blood vessels to maintain water homeostasis is key to survival. Water is lost through respiration, sweating, defecation, and urination and can be saved through glucose metabolism, drinking water, and reabsorbing urine (Boone and Deen, 2008). The kidney is a complex organ of the urinary system comprising glomerular, tubular, mesangial, endothelial, and podocyte cells (Mihevc et al., 2020). The kidney’s collecting duct plays an essential role in maintaining electrolyte balance, acid-base balance, and fluid homeostasis (Palmer and Schnermann, 2015; Carrisoza-Gaytan et al., 2016; Rein et al., 2020).

In proximal tubule bicarbonate reclamation pathways, kidneys maintain the acid-base balance by reabsorption of HCO_3_^−^ and secretion of H^+^. HCO_3_^−^ is reabsorbed by proximal tubular cells in the kidney using the apical Na^+^/H^+^ exchanger (*NHE3*) and the basolateral Na^+^/HCO_3_^−^ cotransporter *NBCe1* (encoded by *SLC4A4*). Moreover, *NBCe1* can make Na^+^ dependent HCO_3_^−^ outflow (Orlowski et al., 2013). Na-K-ATPase plays a vital role in the proximal tubule bicarbonate reclamation pathway. In the kidney, Na-K-ATPase is the essential Na^+^ transporter in the basolateral membrane, and its primary function is to transport Na^+^ out of cells and K^+^ into cells (Horisberger, 2004). Na-K-ATPase lowers intracellular Na^+^ concentration, and thereby providing the driving force for water molecules and sodium uptake across the apical membrane (Vallon and Thomson, 2020). Maintaining the transport system of Na^+^ and K^+^ gradients across the plasma membrane plays a vital role in Na^+^ transport in proximal tubules (Feraille and Doucet, 2001). *AQP1* is in the apical and basolateral plasma membrane of proximal convoluted tubule (PCT), descending limb, and descending vasa recta, mediating water reabsorption (Chou et al., 1999). Mice with *AQP1* deficiency have been observed to be characterized by polyuria, indicating that *AQP1* plays a vital role in hyperpermeability (Yang et al., 2001).

In the vasopressin-regulated water reabsorption pathway, the antidiuretic hormone vasopressin (*AVP*) is one of the primary hormones that regulates renal water permeability. *AVP* released from the pituitary binds to arginine vasopressin receptor 2 (*AVPR2*) in the basolateral membrane of collecting duct principal cells, initiating a signaling transduction cascade to activate adenylate cyclase (*AC*). It catalyzes the conversion of adenosine triphosphate (ATP) to cyclic adenosine monophosphate (*cAMP*), and the intracellular *cAMP* level increases (Tong et al., 2016). The *cAMP*-dependent protein kinase A (*PKA*) is activated, which phosphorylates aquaporin 2 (*AQP2*) and transfers it to the apical plasma membrane rendering the membrane permeable to water (Nielsen et al., 1993; Nielsen et al., 1995). Water is reabsorbed at the apical membrane by *AQP2* and outflow from the basolateral membrane by *AQP3* and *AQP4*, transporting water from urine in the renal tubules to the interstitium and concentrating urine. *PKA* also promotes the expression of *cAMP*-response element-binding protein 3(*CREB3*) in the nucleus, and *CREB3* is located upstream of the *AQP2* transcription start site. As a potential *AQP2* transcription factor binding site, it may indirectly regulate the transcription of the *AQP2* gene (Jung et al., 2018). The *AQP2* protein is located in the apical plasma membrane and the subapical vesicles in collecting-duct principal cells. As the main protein reabsorbing water in the kidney, *AQP2* protein is expressed in the kidneys of humans and other animals, including *L. yarkandensis*, quails, and chickens (Zhang et al., 2019a; Gao et al., 2020; Yang et al., 2021)

Transcription factors (TF) are essential regulatory proteins in eukaryotes, which regulate the spatio-temporal expression of downstream genes. Hypoxia-inducible factor-1 (*HIF-1*) is an oxygen-dependent transcriptional activator that plays an important role in tumor angiogenesis and mammalian development. HIF-1alpha (*HIF1A*) stimulates the expression and activation of glycolytic isomerase that differs from normal cells, thereby supporting tumor-related metabolism by enhancing macromolecular biosynthesis and energy production pathways in human cancers (Marin-Hernandez et al., 2009). High expression of *HIF1A* is associated with high mortality in patients with breast, ovarian, uterine, cervical, brain, and oropharyngeal cancers (Lee et al., 2004). There is evidence that *HIF1A* can directly regulate and inhibit the expression of Collagen 4 subunit A2 (*COL4A2*) in renal cells, which can be used to treat acute kidney injury and protect against chronic kidney disease (CKD) (Sanaei-Ardekani et al., 2021).

Nuclear factor-kappa (*NF-κB*) is a transcription factor family of protein complexes that regulate DNA transcription, signal transduction, and cytokine expression. *NF-κB* is also a key regulator of inflammatory responses (Hayden et al., 2006). *NF-κB* and its inhibitors play a crucial role in the pathogenesis of cancer, kidney disease, and diabetic nephropathy as regulators of the expression of many pro-inflammatory genes (He et al., 2010). In the kidney, NF-κB also binds to upstream *AQP2* transcription sites, thereby inhibiting *AQP2* transcription and regulating renal water reabsorption capacity (Hasler, 2009). The nuclear factor of activated T-cells, cytoplasmic 1 (*NFATc1*), is a transcription factor activated by T-cell receptor (TCR) and Ca_2_^+^ signaling, affecting T-cell activation and effector function. It plays a key role in regulating early gene transcription by T-cell receptor-mediated signaling (Crabtree and Olson, 2002; Hogan et al., 2003; Macian, 2005). Studies have shown that hypertonic stress responses can promote nuclear translocation of the *NFATc* protein and subsequently induce *AQP2* expression (Li et al., 2007).

Much remains to be determined at the genomic level about desert animals. Wu et al. (2014) found that the unique hyperglycemia of camels may be an adaptation to desert life. Ababaikeri et al. (2020) used whole-genome sequencing and analysis of Tarim red deer (*Cervus elaphus yarkandensis*) to identify the genetic basis of high-temperature tolerance (Ababaikeri et al., 2020). The rapid adaptation of sheep (*Ovis aries*) to extreme environments (highlands, deserts) is related to the hypoxia response in plateau environments, water reabsorption in desert environments, and energy metabolism and body size in both environments (Yang et al., 2016). In this research, we used transcriptome sequencing to study the genes and regulatory pathways of water reabsorption in the *L. yarkandensis* kidney based on upstream transcription factors that regulate the expression of target genes. Understanding kidney water reabsorption genes and transcription factors of *L. yarkandensis* can provide insight into molecular mechanisms of drought tolerance.

## Materials and Methods

### Ethics Statement

All animal procedures were approved by the Animal Care and Use Committee of Xinjiang Uygur Autonomous Region, China, and were conducted in accordance with the guidelines developed by the China Council on Animal Care and Protocol.

### Experimental Animals and Tissue Collection

Adult *L. yarkandensis* and *O. cuniculus* were selected and collected in December 2019. Six *L. yarkandensis* was collected from Shaya County, Aksu Prefecture, northwest of the Tarim Basin. And animals were assessed to be adults based on a skull length of greater than 75.50 mm. The average age of 6–8 months and weight of 1.5–1.8 kg. The Animal Laboratory of Tarim University provided six healthy adult rabbits of similar age. These animals were maintained initially in individual cages and had free access to food and drinking water at all times. One week after feeding, the animals were anaesthetized with 20% Ulatan (ethyl carbamate) (0.4 ml/kg) and sacrificed immediately after coma. The kidney was quickly dissected and cut into pieces. The right renal cortex, outer medulla and inner medulla were removed for analyzing the kidney protein, RNA, and the renal cortex for the RNA sequencing. The left kidneys were dissected partially to obtain the cortex, outer medulla and inner medulla, infused with 4% paraformaldehyde (Sigma-Aldrich, Shanghai, China) and then fixed overnight for histologic examination.

### Total RNA Extraction and Quantification

Total RNA-seq using the Trizol method and its experimental protocol were utilized. We used GoodView to detect the quality of RNA dyeing on a 1% agarose gel and used nucleic acid protein tester BioSpectrometer (Eppendorf) determination of purity of RNA. The RNA was then reversely transcribed into cDNA using EasyScript^®^ One-Step gDNA Removal and cDNA Synthesis SuperMix (Transgen, Beijing, China).

### RNA-Seq Library Construction and Unreferenced Transcriptome Sequencing

A total amount of 1.5 µg RNA per sample was used as input material for the RNA sample preparations. Following the manufacturer’s recommendations, sequencing libraries were generated using NEBNext^®^ Ultra™ R.N.A. Library Prep Kit for Illumina^®^ (NEB, United States), and index codes were added to attribute sequences to each sample. The library fragments were purified using the AMPure XP system (Beckman Coulter, Beverly, United States). Then, 3 µL USER Enzyme (NEB, United States) was used with size-selected, adaptor-ligated cDNA at 37°C for 15 min followed by 5 min at 95°C before PCR. Then PCR was performed with Phusion High-Fidelity DNA polymerase, Universal PCR primers, and Index (X) Primer. PCR products were purified (AMPure XP system), and library quality was assessed on the Agilent Bioanalyzer 2100 system.

### De Novo Assembly With Functional Annotations

Raw data (raw reads) of fastq format were first processed through in-house Perl scripts. Clean data (clean reads) were obtained by removing reads containing adapters, ploy-N, and low-quality reads from raw data. Q20, Q30, GC-content, and sequence duplication levels of the clean data were calculated. All downstream analyses were based on clean data with high quality. High-quality RNA sequencing data from the library were assembled using Trinity software. If the same genes identified multiple transcripts, we chose one of the longest transcripts as representative of the gene sequence, hereafter referred to as the unigene sequence.

All unigene sequences were searched for functional annotations in several databases: Nr (NCBI non-redundant protein sequences); Nt (NCBI non-redundant nucleotide sequences); Pfam (Protein family); KOG/COG (Clusters of Orthologous Groups of proteins); Swiss-Prot (a manually annotated and reviewed protein sequence database); KO (KEGG Ortholog database); and GO (Gene Ontology).

### Quantification of Gene Expression and Differential Gene Expression Analysis

The transcriptome spliced at Trinity was used as the reference sequence, and clean reads of each sample were mapped to this. In this process, we used RSEM software (Li and Dewey, 2011) for Bowtie comparisons, obtained the read count number of each sample compared to each gene, and performed Fragments Per Kilobase per Million (FPKM) conversion to analyze the gene expression level.

The read count data were analyzed for gene differential expression using the DEseq R package (1.10.1). DESeq provides statistical routines for determining differential expression using a model based on the negative binomial distribution. The resulting *p* values were adjusted using Benjamini and Hochberg’s approach for controlling the false discovery rate. Genes with an adjusted *p*-value < 0.05 as found by DESeq were assigned as differentially expressed.

### Analysis of Enrichment

Gene Ontology (GO) enrichment analysis of the differentially expressed genes (DEGs) was implemented by the GOseq R package based on the Wallenius non-central hypergeometric distribution (Young et al., 2010) which can adjust for gene length bias in DEGs. We used KOBAS (Mao et al., 2005) software to test the statistical enrichment of DEGs in KEGG pathways. We identified significantly enriched GO terms and signaling pathways (*p* < 0.05).

### Prediction of Transcription Factors and Target Genes

SamTools (http://samtools.sourceforge.net/) and BLASTX in BLAST+ were used to compare unigene sequences to the *O. cuniculus* transcription factor dataset in the AnimalTFDB 3.0 database (http://bioinfo.life.hust.edu.cn/AnimalTFDB) (Hu et al., 2019). BLASTX result files were interpreted and screened, and TF Length >110 amino acids and identity >80% were selected as screening conditions. The TRANSFAC database (https://genexplain.com/transfac/) was adopted to predict the TF corresponding to target gene prediction. Unigene ID exists in each TF and target gene sequence for subsequent analysis.

### Functional Prediction of Renal Water Reabsorption Transcription Factors

The DEG KEGG pathway enrichment analysis results determined that the renal water reabsorption signaling pathways were mainly vasopressin-regulated via the proximal tubule bicarbonate recovery pathway. We took DEGs as the target genes, which play a key role in the pathway. We studied the functional predictions and regulatory analysis of transcription factors upstream of the target gene according to the regulatory relationship between the predicted transcription factors and the target gene.

### Validation of RNA-Seq Experiments

To verify the accuracy and validity of transcriptome sequencing data, reverse transcription cDNA products were amplified by polymerase chain reaction (PCR). Primers targeted *AQP1* (Cluster-1445.13691), *AQP2* (Cluster-1445.18746), *CREB3* (Cluster-1445.10450), *ADCY3* (Cluster-1445.26438), *FXYD2* (Cluster-1445.18046), *HIF1A* (Cluster-1445.20583), and *NFATc1* (Cluster-1445.8040). (Supplementary Table S1). Polymerase chain reactions consisted of 10 μM of each primer, dNTP Mixture, and TB Green^®^ Premix Ex TaqTM Ⅱ (Takara Bio, Beijing, China), in a total reaction volume of 20 μl. Quantitative RT-PCR was performed on a CFX96 Touch Deep Well (Bio-Rad, Delaware, American). Conditions were 95°C for 3 min, 45 cycles of 95°C for 5 s, 60°C for 30 s, and 72°C for 10 s. Analysis of relative gene expression was performed using the 2^−ΔΔCt^ method (Schmittgen and Livak, 2008). Values were normalized to *GAPDH* values and expressed as gene/*GAPDH* ratio.

### Protein Extraction and Western Blot

Total proteins were isolated from the kidneys of *O. Cuniculus* and *L. yarkandensis*. A 100 mg sample of right kidney tissue was isolated and placed in chilled lysis buffer containing 0.04 M Tris-HCl (PH 7.4), 0.82% NaCl, 1.5% Triton X-100, 0.5% deoxycholic acid sodium salt, 0.1% SDS, a protease inhibitor cocktail (Sigma-Aldrich, Shanghai, China), and 1 mM PMSF. The tissues were homogenized in 1 ml of lysis buffer. The homogenates were placed on ice for 20 min and then were centrifuged at 12,000 × g for 20 min at 4°C. The supernatants were then collected. The total protein concentration was measured using a BCA protein assay reagent kit (Aidlab, Beijing, China) according to the manufacturer’s protocol.

The total proteins were solubilized in Laemmli sample buffer at 70°C for 8 min and then were subjected to SDS-polyacrylamide gel electrophoresis. After transferring to polyvinylidene difluoride (PVDF) membranes (Bio-Rad, Delaware, American), western blotting membranes were stripped and blocked, followed by incubation overnight at 4 °C with antibodies against *HIF1A*, *NFATC1*, *NF-κB1*, *ADCY3*, *ATP1A1*, *SLC4A4* and *GAPDH* (Proteintech, Wuhan, China). After washing, the membranes were incubated with horseradish peroxidase (HRP)-labeled anti-rabbit secondary antibody (Proteintech, Wuhan, China) for 1 h at room temperature and then were visualized via enhanced chemiluminescence (SuperSignal, Pierce). Western blotting was scanned using Tanon 5200, and then the labeling density was quantified using Gel-Pro Analyzer4. Integral optical density (IOD) was used to represent the density measurement results of the target protein bands, and then the determination results of the target protein and corresponding *GAPDH* protein were normalized. Finally, the mean values of *L. yarkandensis* and *O. cuniculus* were compared.

### Immunohistochemistry

After dissecting, the kidney tissues of *L. yarkandensis* and *O. cuniculus* were fixed in 4% paraformaldehyde. For histological analysis, the cortex, outer medulla and inner medulla of the left kidney were paraffin-embedded with the usual paraffin embedding method. The paraffin-embedded cortex, outer medulla, and inner medulla were sectioned at 6 µm for immunohistochemical staining of *ADCY3*, *ATP1A1* and *SLC4A4* deparaffinized by washing in xylene three times for 10 min each. This was followed by rehydration through a series of ethanol washes from 100% to 70% ethanol. The slides were then placed in methanol containing 0.5% hydrogen peroxide for removing the endogenous peroxidase activity. Non-specific binding was blocked by incubating the slides for 1 h at room temperature in 5% BSA. The cortex, outer medulla, and inner medulla sections were incubated with antibodies against *ADCY3* (Proteintech, 19492-1-AP, 1:100), *ATP1A1* (Proteintech, 14418-1-AP, 1:240), and *SLC4A4* (Proteintech, 11885-1-AP, 1:500) overnight at 4°C. The sections were rinsed in 0.1 M PBS (PH 7.2–7.4) and then incubated for 30 min at room temperature with HRP-labeled goat anti-rabbit secondary antibody (Proteintech, Wuhan, China). The sections were washed with PBS, incubated with diaminobenzidine for 6 min, and then washed again. The tissue sections were stained using hematoxylin (Sigma-Aldrich, Shanghai, China) for 40 s and then washed under running water for 5 min. The primary antibody was substituted with PBS, and the controls underwent a similar procedure as described before. The immunostained sections were observed and photographed on a microscope (Motic BA600-4, Beijing, China). The captured images were analyzed in the IpWin32 software for each group’s IOD and area of immunopositive cells. The difference in the average density (IOD/Area) between the groups was compared.

### Statistical Analysis

Statistical analysis software Graphpad Prism was used to calculate the means and standard errors. A student’s t-test was used to assess the differences between groups. A *p*-value of less than 0.05 was considered statistically significant.

## Results

### Transcriptome Data Analysis

We constructed six samples containing three biologically repetitive cDNA libraries from *O. cuniculus* and *L. yarkandensis*. We sequenced the libraries using an Illumina high-throughput sequencing platform. The six samples were named OC_1, OC_2, and OC_3 (*O. cuniculus*), and LY_1, LY_2, and LY_3 (*L. yarkandensis*). For the sequencing data of these 6 samples, Raw Reads were filtered to remove the low-quality data. The remaining clean reads data showed little difference, and the readings were 21.92, 20.22, 21.30, 19.94, 21.86, and 21.93 million, respectively (Supplementary Table S2). The sequences based on clean reads were analyzed and spliced by Trinity. The longest transcript of each gene was selected as the unigene sequence for a total of 42,998 unigene sequences of *L. yarkandensis*; 15,413 unigene sequences ranged from 300 to 500 bp and 16,648 unigene sequences exceeded 1,000 bp (Supplementary Table S3, Figure S1).

To obtain the potential functional information of unigene sequences, we searched and annotated seven databases, including Nr, Nt, Pfam, KOG/COG, Swiss-Prot, KEGG, and GO. The proportion of unigene sequences on annotations ranged from 10.76% to 78.84%. Among the 42,998 unigene sequences, NR and NT databases annotated 20,010 (46.53%) and 33,038 (76.83%) sequences (Supplementary Table S4).

### Functional Classification of Unigenes

**FIGURE 1:**
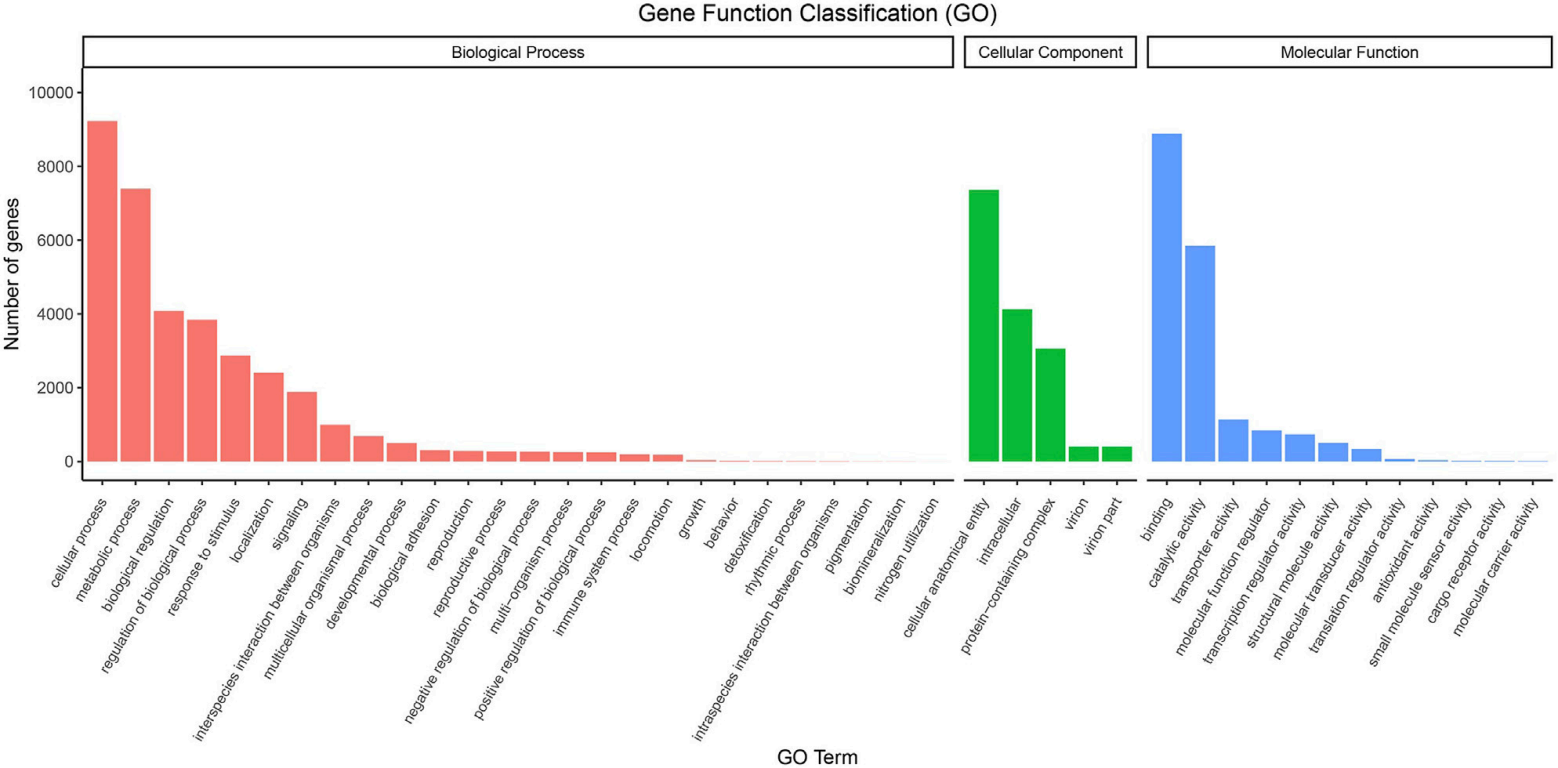
GO classifications of the unigene sequences. Annotated unique sequences were classified into Biological Process, Cellular Component, and Molecular Function.

We used GO Terms and KEGG Pathways to analyze the functionality of the unigenes; 15,263 unigenes were annotated with GO terms. These belong to 43 functional groups, distributed in three main categories: molecular function, biological process, and cellular component (Figure 1). In the biological process category, cellular process, metabolic process, biological regulation, and regulation of biological process are the functions of many genes. In the cellular component category, intracellular, cellular anatomical entity, and protein-containing complex are the main functions. Among the molecular functions, binding, catalytic activity, and transporter activity annotate the functions of many genes (Supplementary Table S5).

**FIGURE 2:**
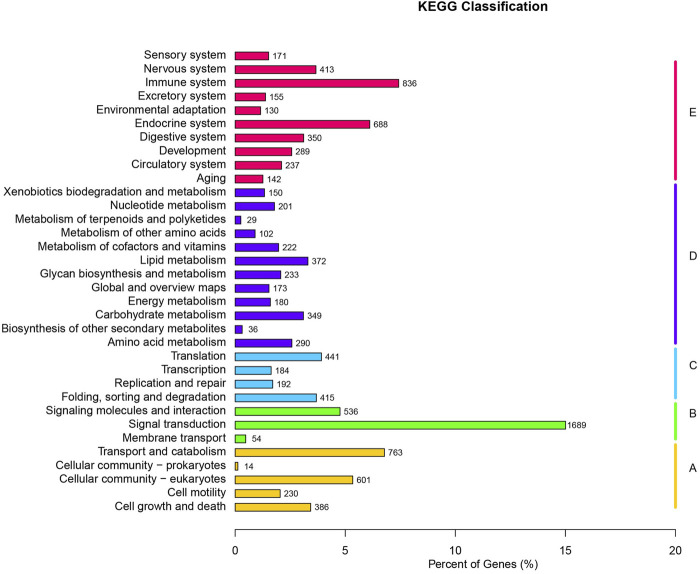
KEGG classifications of all unique sequences. **(A)** Cellular Processes; **(B)** Environmental Information Processing; **(C)** Genetic Information Processing; **(D)** Metabolism; **(E)** Organismal Systems.

For KEGG Pathways, 11,253 unigenes were annotated. The top 10 pathways are signal transduction (1,689 sequences), immune system (836 sequences), transport and catabolism (763 sequences), endocrine system (688 sequences), Cellular community-eukaryotes (601 sequences), signaling molecules and interaction (536 sequences), translation (441 sequences), folding, sorting and degradation (415 sequences), and nervous system (413 sequences) (Figure 2). KEGG analysis shows that 26 unigene sequences are proximal tubule bicarbonate reclamation pathways; 48 unigene sequences are involved in vasopressin-regulated water reabsorption pathways.

### Determination of Gene Abundance and Identification of Differentially Expressed Genes

**FIGURE 3:**
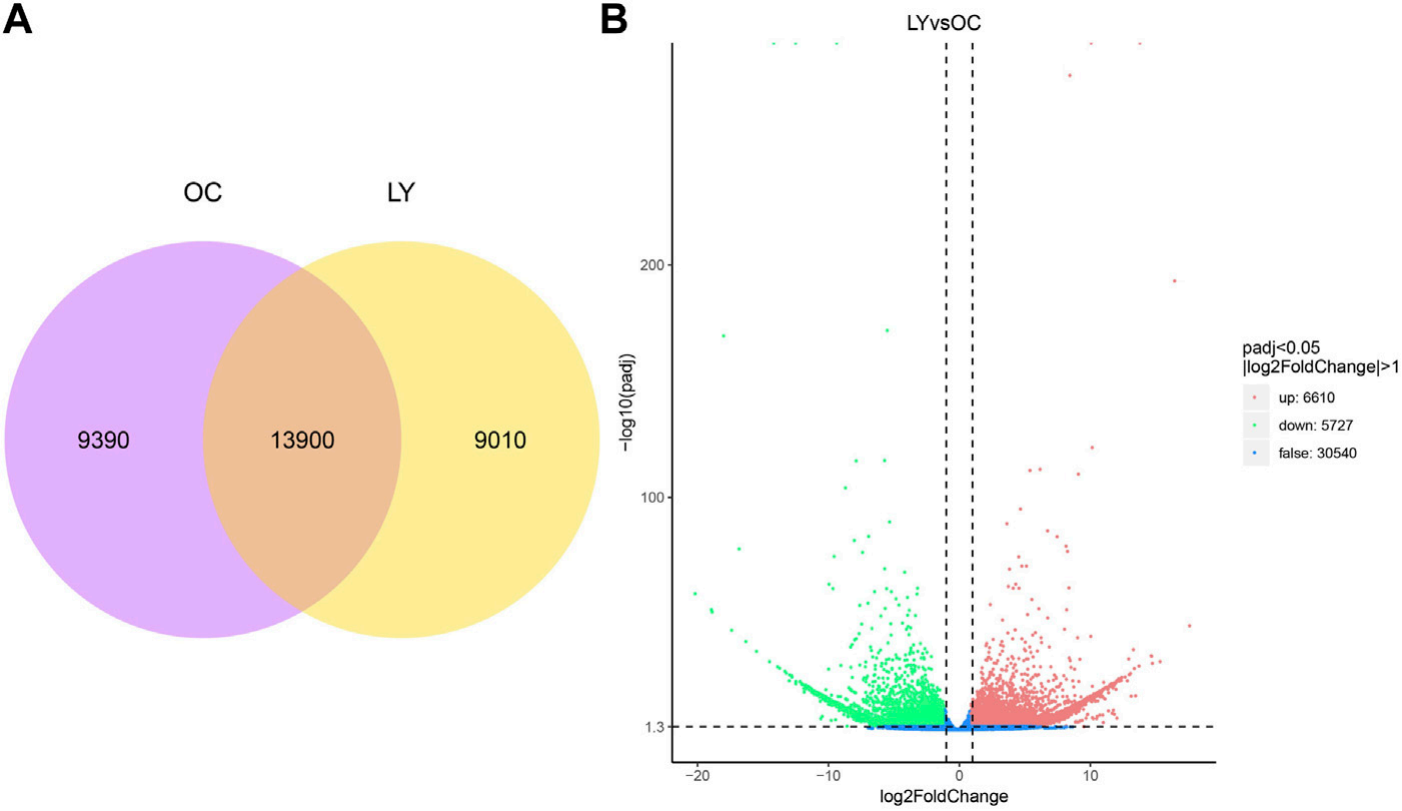
Determination of gene expression level and identification of differentially expressed genes. Venn diagram indicating the numbers of genes expressed under different species **(A)**. OC, *O cuniculus*; LY, *L. yarkandensis.* Volcano diagram of gene differential expression analysis under different species **(B)**. The abscissa represents the gene expression multiple, and the ordinate represents the statistical significance of gene expression changes. The lower the corrected *p*-value, the larger the -log10 (corrected *p*-value) is. The more significant the difference is. The dots in the figure represent genes, with blue dots representing genes with no significant difference, red dots representing genes with significant difference up-regulation, and green dots representing genes with significant down-regulation.

Gene expression levels of unigene sequences were identified and expressed by FPKM. Our study used two species treatment groups to analyze the ReadCount of gene expression level analysis. *L. yarkandensis* and *O. cuniculus* had 22,910 and 23,290 expressed genes, respectively. Among them, 9,010 and 9,390 genes were unique to *L. yarkandensis* and *O. cuniculus*, respectively, and the two species shared 13,900 genes (Figure 3A).

The up-regulation distribution and *p*-value changes of differentially expressed genes (DEGs) are shown in the volcano diagram: 6,610 up-regulated genes and 5,727 down-regulated genes were expressed in *L. yarkandensis* (*p* < 0.05 and |log2FoldChange| > 1) (Figure 3B). These DEGs reflect the significant difference in gene expression level between *L. yarkandensis* and *O. cuniculus*.

### Functional Enrichment Analysis of DEGs

To further determine the range of DEGs related to water reabsorption, DEGs were first positioned using GO Enrichment. The largest difference between *L. yarkandensis* and *O. cuniculus* are intracellular (1,163 DEGs), cellular nitrogen compound metabolic process (1,149 DEGs), ion binding (1,002 DEGs), biosynthetic process (905 DEGs), and organelle (875 DEGs). The results showed that *L. yarkandensis* and *O. cuniculus* differed in fundamental biological processes, including ion binding, intracellular, and biosynthetic process (Supplementary Table S6). Also, 1,559 DEGs were located as KEGG Pathways. The largest differences between *L. yarkandensis* and *O. cuniculus* are pathways in cancer (113 DEGs), PI3K-Akt signaling pathway (111 DEGs), focal adhesion (87 DEGs), regulation of actin cytoskeleton (68 DEGs), rap1 signaling pathway (62 DEGs), and endocytosis (60 DEGs). The results showed that *L. yarkandensis* and *O. cuniculus* had different expression levels in cancer, actin cytoskeleton, and some biological tissues (Supplementary Table S7).

### Transcription Factors and Water Reabsorption

According to BLAST comparison results of unigenes and the *O. cuniculus* transcription factor database. We predicted 1,159 transcription factors from unigene, of which 232 transcription factors were differentially expressed (Supplementary Table S8).

After obtaining predicted transcription factors, we searched for the Symbol ID of the transcription factor and corresponding downstream target genes of each transcription factor in the TRANSFAC database. Among the 232 differentially expressed transcription factors predicted, 20 existed in the database, and 1,137 corresponding target genes were predicted. Each transcription factor and target gene sequence has a corresponding unigene sequence, which was used for subsequent analysis (Supplementary Table S9).

To study the water reabsorption capacity of *L. yarkandensis*, we studied DEGs regulating water reabsorption via pathways: the vasopressin-regulated water reabsorption pathway and the proximal tubule bicarbonate reclamation pathway. From the two pathways, it was found that five DEGs (*AQP1*, *AQP2*, *CREB3*, *ADCY3*, and *FXYD2*) were significantly up-regulated (4 genes) or down-regulated (1 gene). These DEGs may play a key role in water reabsorption in their respective pathways. By cross-comparing the data of 5 water reabsorption DEGs and transcription factors and their target genes, we obtained related transcription factors that may regulate these 5 differential genes. Since a gene can be predicted to have multiple transcription factors, we selected two DEGs with significant differential expression, namely *HIF1A* and *NFATc1*, as the target transcription factors (Supplementary Table S10).

### mRNA Expression of Water Reabsorption DEGs

**FIGURE 4:**
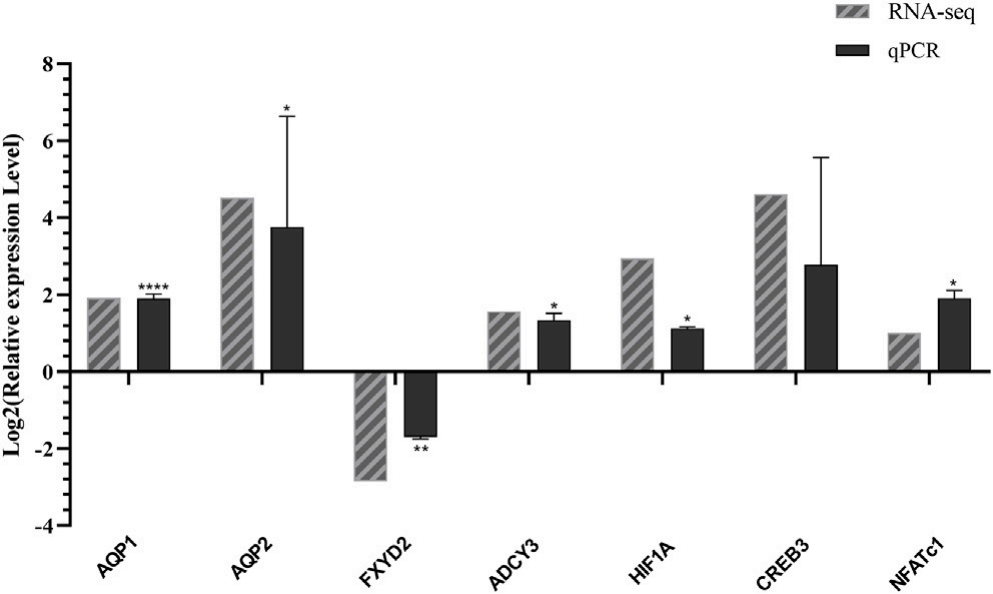
Differentially expressed genes were identified by qPCR. RNA-seq: log_2_FoldChange number of unigenes *L. yarkandensis:O. cuniculus* in transcriptome data; qPCR: Log_2_ value of gene expression level relative to *L. yarkandensis:O. cuniculus* mRNA in RT-qPCR results.

To verify the accuracy and validity of transcriptome sequencing results and the reliability of prediction results of transcription factors and their target genes, we used quantitative RT-PCR to detect the mRNA expression levels. These genes include *AQP1* (Cluster-1445.13691), *AQP2* (Cluster-1445.18746), *CREB3* (Cluster-1445.10450), *ADCY3* (Cluster-1445.26438), *FXYD2* (Cluster-1445.18046), *HIF1A* (Cluster-1445.20583), and *NFATc1* (Cluster-1445.8040). We then compared the expression level of the transcriptome sequencing results and analysis. As shown in Figures 4, 7 genes determined by the RNA-seq experiment had a good correlation with the experimental results of RT-qPCR. Among them, *AQP1*, *AQP2*, *ADCY3*, *CREB3*, *HIF1A*, and *NFATc1* were up-regulated in L. yarkandensis and *FXYD2* was down-regulated. This finding indicates that our RNA-seq sequencing results are reliable.

### Expression of Renal Water Reabsorption Transcription Factors and Target Proteins

According to the analysis of RT-qPCR data, *ADCY3* was significantly up-regulated in the kidney of *L. yarkandensis* compared with *O. cuniculus*. Literature has shown that *AQP2* expression level is related to transcription factor *NF-κB1* (Albertoni Borghese et al., 2018). In the proximal tubule bicarbonate reclamation pathway, *ATP1A1* and *SLC4A4* proteins also play a critical role. In order to further analyze the regulatory mechanism of the proximal tubule bicarbonate reclamation pathway and vasopressin-regulated water reabsorption pathway, protein abundance of *SLC4A4*, *ATP1A1* and transcription factor *NF-κB1* were measured by western blot.

**FIGURE 5:**
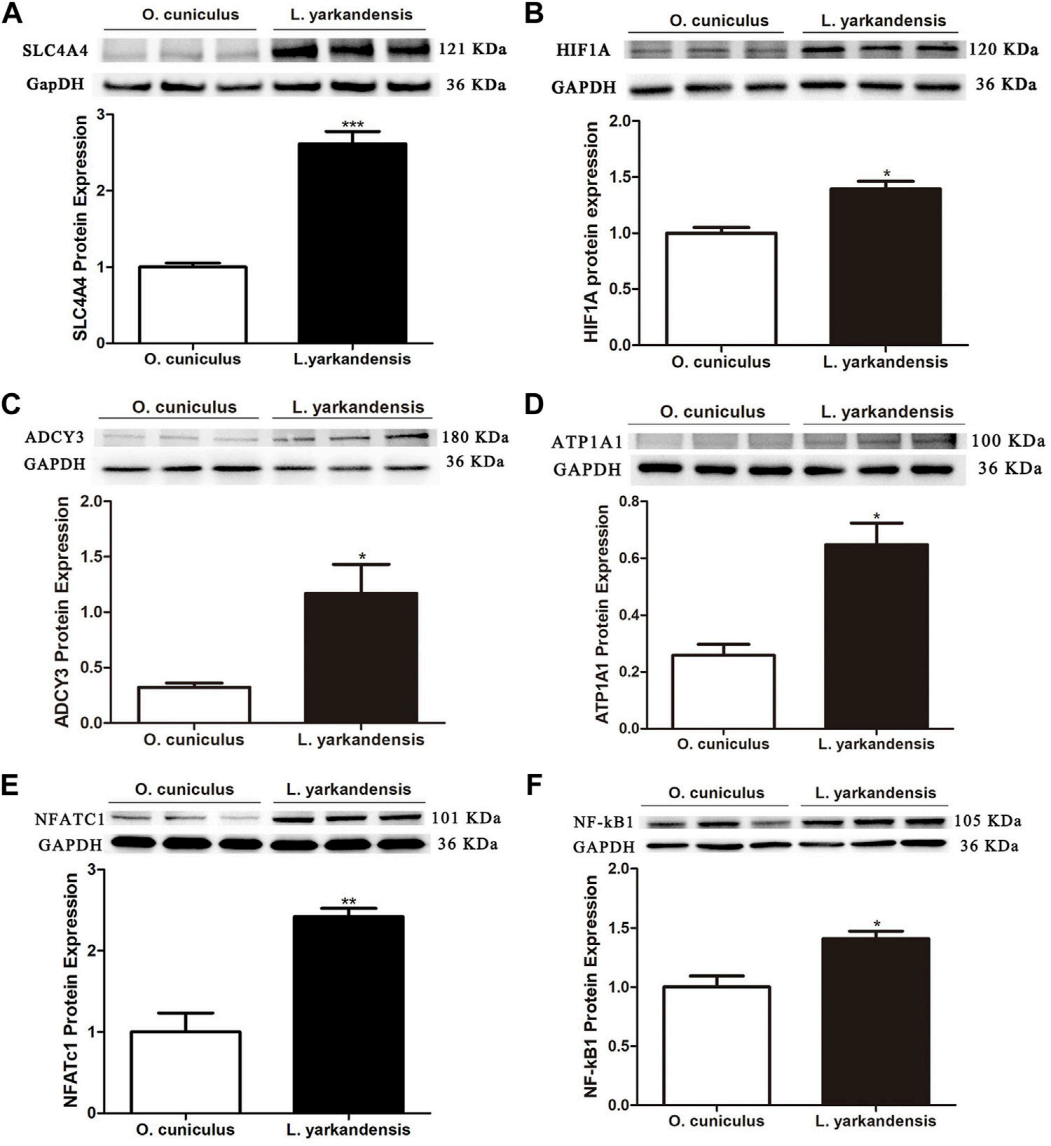
Protein expression in kidney of *O. cuniculus* and *L. yarkandensis*. **(A)** (Top) Representative western blotting analyses of *SLC4A4* protein expression in the renal of *O. cuniculus* and *L. yarkandensis*. Immunoblots of total proteins were probed with anti-SLC4A4 antibody and identified 121 kDa bands, while GAPDH antibody identified 36 kDa bands. (Bottom) Densitometry of all western blotting results of the renal of *O. cuniculus* and *L. yarkandensis* (n = 6 for each group); **(B)** (Top) Representative western blotting analyses of *HIF1A* protein expression in the renal of *O. cuniculus* and *L. yarkandensis*. (Bottom) Densitometry of all western blotting results of the renal of *O. cuniculus* and *L. yarkandensis* (n = 6 for each group); **(C)**: (Top) Representative western blotting analyses of *ADCY3* protein expression in the renal of *O. cuniculus* and *L. yarkandensis*. (Bottom) Densitometry of all western blotting results of the renal of *O. cuniculus* and *L. yarkandensis* (n = 6 for each group); **(D)**: (Top) Representative western blotting analyses of *ATP1A1*protein expression in the renal of *O. cuniculus* and *L. yarkandensis*. (Bottom) Densitometry of all western blotting results of the renal of *O. cuniculus* and *L. yarkandensis* (n = 6 for each group); **(E)**: (Top) Representative western blotting analyses of *NFATC1*protein expression in the renal of *O. cuniculus* and *L. yarkandensis*. (Bottom) Densitometry of all western blotting results of the renal of *O. cuniculus* and *L. yarkandensis* (n = 6 for each group); **(F)**: (Top) Representative western blotting analyses of *NF-kB1*protein expression in the renal of *O. cuniculus* and *L. yarkandensis*. (Bottom) Densitometry of all western blotting results of the renal of *O. cuniculus* and *L. yarkandensis* (n = 6 for each group); The histogram shows optical density analysis of western blot results, for ^∗∗∗^ *p* < 0.001, ^∗∗^ *p* < 0.01, and ^∗^ *p* < 0.05.

To further analyze whether the protein abundance of *ADCY3*, *ATP1A1*, and *SLC4A4*, and transcription factors *HIF1A*, *NFATc1*, and *NF-κB1*, western blot was used to detect the protein expression levels of *ADCY3*, *ATP1A1*, *SLC4A4*, *HIF1A*, *NFATc1*, and *NF-κB1* in the kidney of both *L. yarkandensis* and *O. cuniculus*. As shown in Figure 5, *GAPDH* was used as the internal reference protein, and there was a band at 36 KDa incubated with a *GAPDH* antibody. There was a band at 121 KDa when *SLC4A4* antibody was cultured, and integral optical density (IOD) analysis of western blot results showed that the protein expression level of *SLC4A4* in the kidney of *L. yarkandensis* was significantly up-regulated (*p* < 0.001) (Figure 5A). The *HIF1A* antibody was incubated with a band at 120 kDa, and IOD analysis showed that the *HIF1A* protein expression level in the kidney of *L. yarkandensis* was up-regulated (*p* < 0.05) (Figure 5B). The *ADCY3* antibody was incubated with a band at 180 kDa, and IOD analysis showed that the *ADCY3* protein expression level in the kidney of *L. yarkandensis* was up-regulated (*p* < 0.05) (Figure 5C). The *ATP1A1* antibody was incubated with a band at 100 kDa, and IOD analysis showed that the *ATP1A1* protein expression level in the kidney of *L. yarkandensis* was up-regulated (*p* < 0.05) (Figure 5D). The *NFATc1* antibody was incubated with a band at 101 kDa, and IOD analysis showed that the *NFATc1* protein expression level in the kidney of *L. yarkandensis* was up-regulated (*p* < 0.01) (Figure 5E). The *NF-κB1* antibody was incubated with a band at 105 kDa, and IOD analysis showed that the *NF-κB1* protein expression level in the kidney of *L. yarkandensis* was up-regulated (*p* < 0.05) (Figure 5F) (Supplementary Figure S2).

### Distribution of Water Reabsorption Protein

**FIGURE 6:**
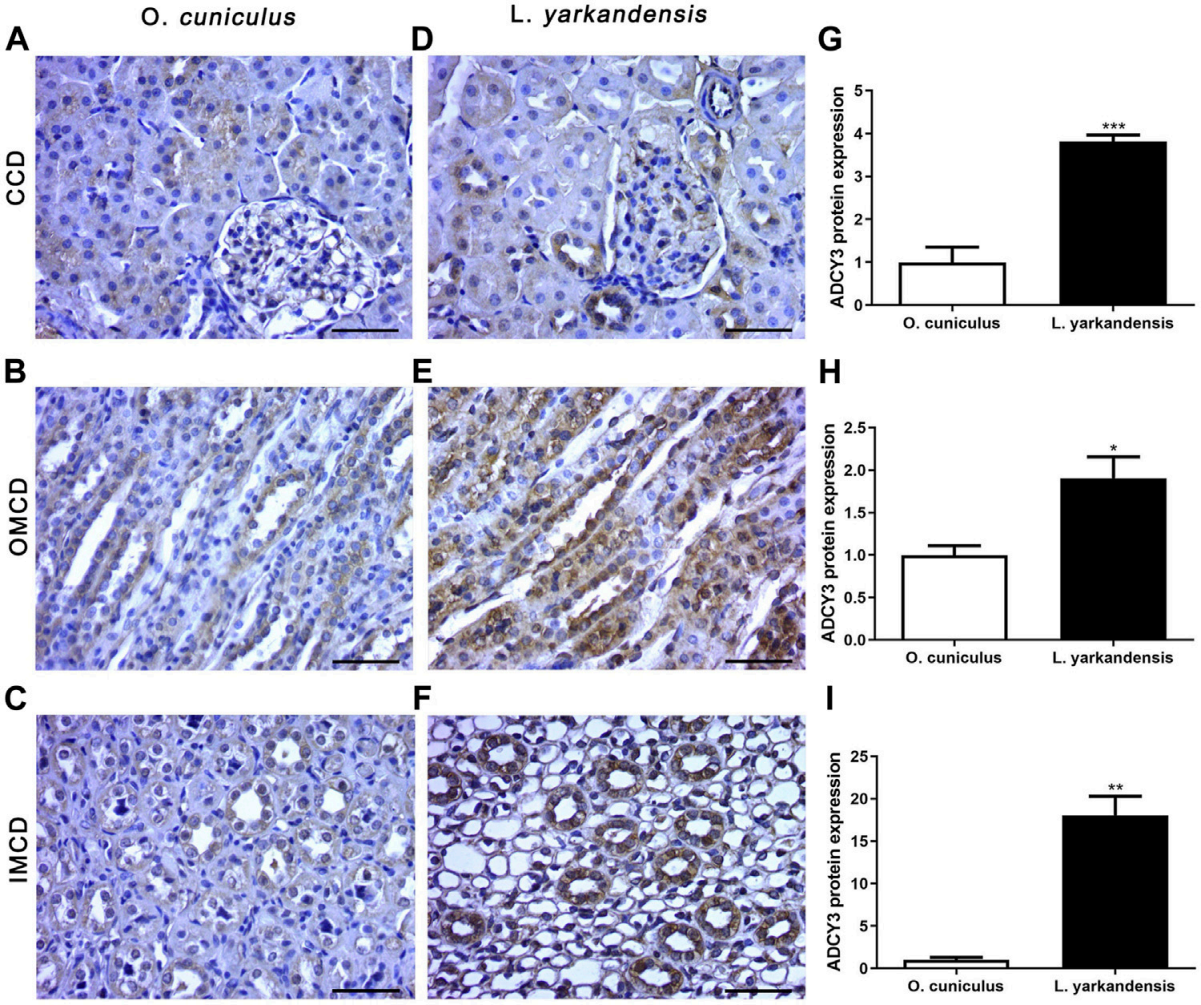
*ADCY3* protein distribution in renal CCD, OMCD, IMCD in a tissue section of *O. cuniculus*(A-C) and *L. yarkandensis* (D-F). Paraffin sections (6 µm) of renal cortex and medulla of *O. cuniculus* and *L. yarkandensis* **(A-F)**. The sections were incubated with an anti-*ADCY3* antibody scale bar 50 µm. Densitometry of all immunohistochemistry results of renal CCD and OMCD and IMCD from *O. cuniculus* and *L. yarkandensis* (n = 6 for each group), ^∗∗∗^ *p* < 0.001, ^∗∗^ *p* < 0.01, and ^∗^ *p* < 0.05 **(G-I)**. Average density showed a significant higher expression of *ADCY3* protein abundance in CCD and OMCD and IMCD of *L. yarkandensis* compared to that of *O. cuniculus* **(G-I)**.

Immunohistochemistry data show that positive signals of the *ADCY3* protein are mainly located in the basolateral membrane of collecting duct cells, and positive staining is light yellow or brownish-yellow. IHC mean optical density analysis showed that compared with *O. cuniculus*, the *ADCY3* protein was strongly labeled in the basolateral membrane of the renal cortical collecting duct (CCD) of *L. yarkandensis* (Figures 6A,D), with significantly up-regulated expression (*p* < 0.001). Strong markers were detected in the basolateral membrane of the outer medullary collecting duct (OMCD) (Figures 6B,E), which was significantly up-regulated (*p* < 0.05). Strong markers were detected in the basolateral membrane of the renal inner medullary collecting duct (IMCD) (Figures 6C,F), presenting an extremely significant up-regulation (*p* < 0.01) (Figure 6).

**FIGURE 7:**
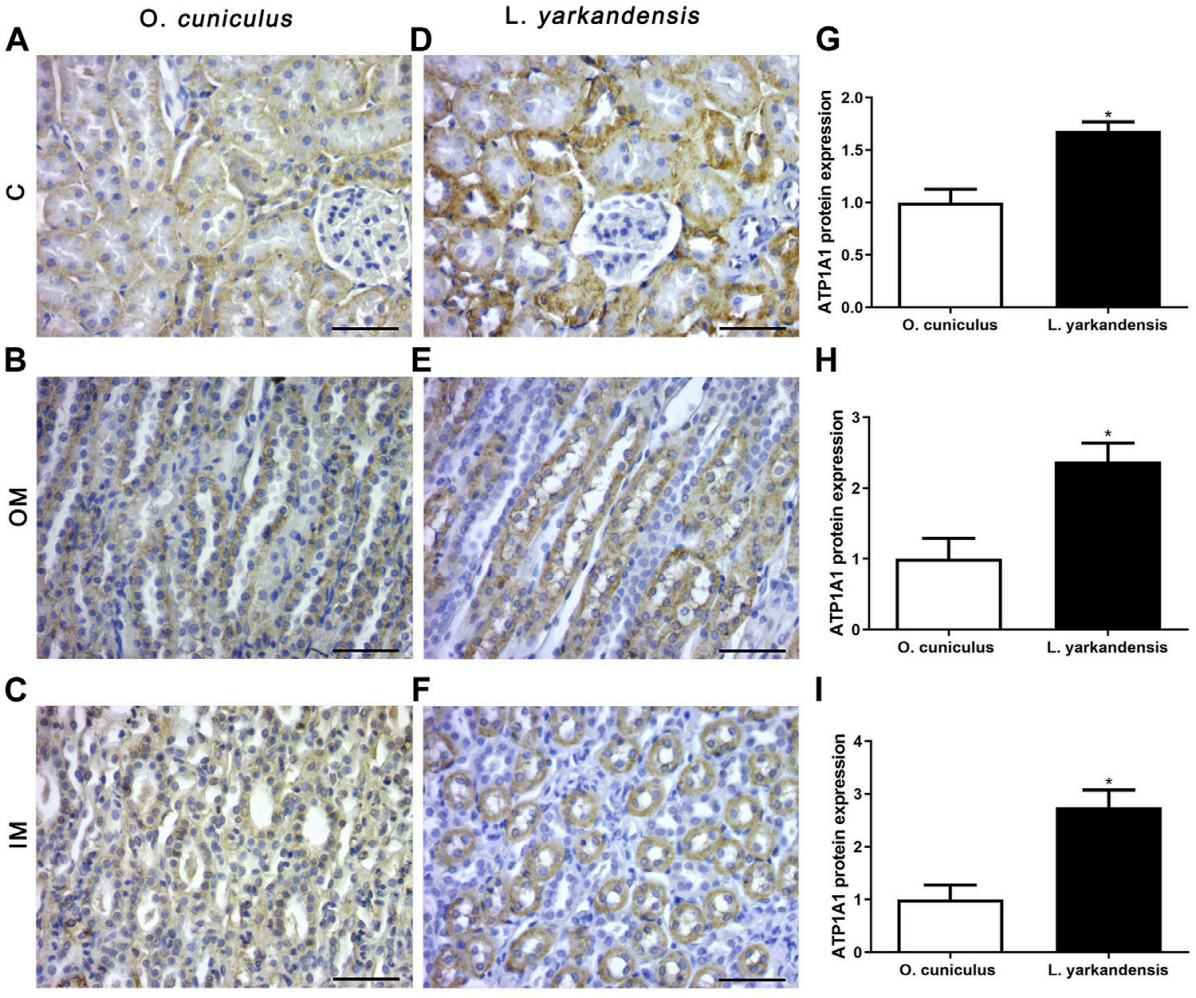
*ATP1A1* protein distribution in renal C, OM, IM in a tissue section of *O. cuniculus* and *L. yarkandensis*. Paraffin sections (6 µm) of renal cortex and medulla of *O. cuniculus* and *L. yarkandensis* **(A-F)**. The sections were incubated with an anti-*ATP1A1* antibody scale bar 50 µm. Densitometry of all immunohistochemistry results of renal C and OM and IM from *O. cuniculus* and *L. yarkandensis* (n = 6 for each group), ^∗^ *p* < 0.05 **(G-I)**. Average density showed a significant higher expression of *ATP1A1* protein abundance in C and OM and IM of *L. yarkandensis* compared to that of *O. cuniculus* **(G-I)**.

As shown in Figure 7, the IHC positive signals of *ATP1A1* protein are located in the proximal basolateral membrane of the PCT and the thin limbs (TL) of the renal medulla, and the positive staining is light yellow or brown-yellow. Compared with *O. cuniculus*, strong markers of *ATP1A1* protein were detected in the basolateral membrane of PCT in the renal cortex of *L. yarkandensis* (Figures 7A,D), and their expression was significantly up-regulated (*p* < 0.05). Strong markers were detected in the basolateral membrane of the proximal tubule straight of OM in *L. yarkandensis* (Figures 7B,E) and they were significantly up-regulated (*p* < 0.05). Strong markers were detected in the TL of the inner medulla in *L. yarkandensis* (Figures 7C,F) and were significantly up-regulated (*p* < 0.05).

**FIGURE 8:**
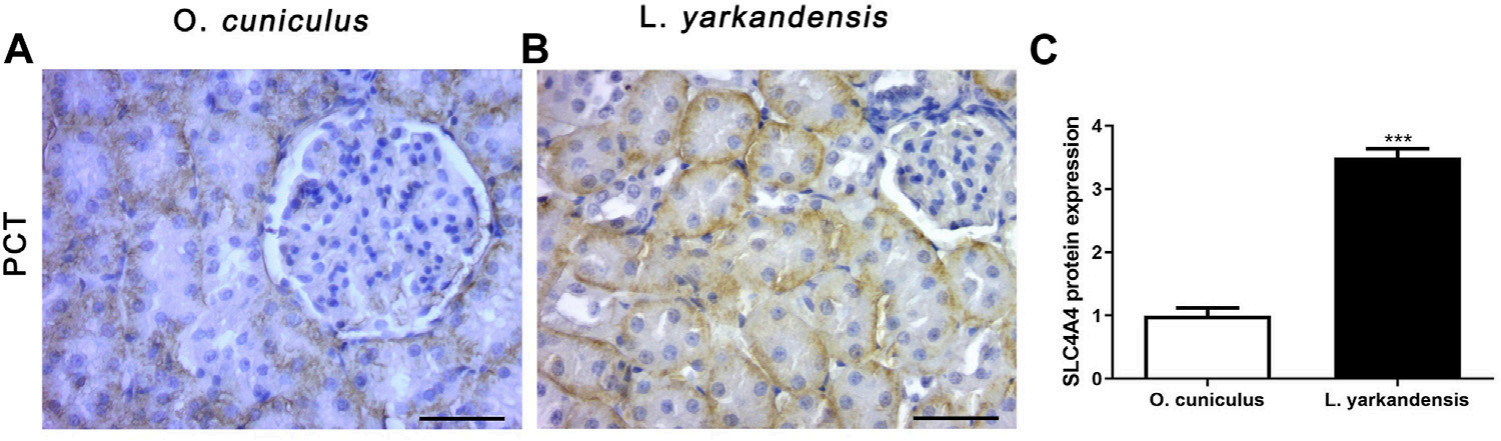
*SLC4A4* protein distribution in renal PCT in a tissue section of *O. cuniculus* and *L. yarkandensis*. Paraffin sections (6 µm) of renal cortex and medulla of *O. cuniculus* and *L. yarkandensis* **(A-B)**. The sections were incubated with anti-*SLC4A4* antibody scale bar 50 µm. Densitometry of all immunohistochemistry results of renal PCT from *O. cuniculus* and *L. yarkandensis* (n = 6 for each group), ^∗∗∗^ *p* < 0.001**(C)**. Average density showed a significant higher expression of *SLC4A4* protein abundance in PCT of *L. yarkandensis* compared to that of *O. cuniculus* **(C)**.

As shown in Figure 8, the positive IHC signal of *SLC4A4* protein was in the basolateral membrane of PCT, and the positive staining was light yellow or brownish-yellow. Compared with *O. cuniculus*, the *SLC4A4* protein was strongly labeled in the basolateral membrane of PCT in the kidney of *L. yarkandensis* (Figures 8A,B) and was significantly up-regulated (*p* < 0.001). However, in the staining of the renal medulla, *SLC4A4* was not expressed in *O. cuniculus* and *L. yarkandensis*. The gene was only expressed in the PCT basolateral membrane of the renal cortex.

## Discussion

### Transcriptome Analysis of the Kidney of *L. Yarkandensis*

According to transcriptome sequencing data and DEGs analysis results, *L. yarkandensis* and *O. cuniculus* shared 42,998 unigene sequences, including 6,610 genes were significantly up-regulated, 5,727 genes were significantly down-regulated, and 30,540 genes were not differentially expressed. GO enrichment analysis and KGEE pathway enrichment analysis found that many essential cell functions, biological processes, and pathways of organisms have different degrees of gene expression. According to the sequence analysis of DEGs, 232 differentially expressed transcription factors were identified, among which 5 genes and 7 transcription factors were found in relation to both vasopressin-regulated water reabsorption pathways and proximal tubule bicarbonate reclamation pathways. According to the difference in expression of transcription factors, gene screening and literature support, 3 transcription factors and 7 genes were selected to experiment.

### Genes and Transcription Factors Regulating Water Reabsorption

In proximal tubule bicarbonate reclamation pathway, previous studies have shown that the primary function of the *AQP1* proximal renal tubule is to absorb more than 70% of the water from the glomerular filtrate (Katsura et al., 1995). The expression levels of *AQP1* and *AQP2* in the kidneys of *L. yarkandensis* were higher than those of *O. cuniculus*, which may be related to the increased water permeability of renal tubules of *L. yarkandensis* (Zhang et al., 2019a). Therefore, the high expression of *AQP1* in *L. yarkandensis* will likely increase the permeability of renal tubules to water. Quantitative RT-PCR results of the mRNA level showed that the *AQP1* was up-regulated, and the *FXYD2* was down-regulated. Western blotting showed that the *SLC4A4* protein and *ATP1A1* protein were highly expressed in the kidney of *L. yarkandensis*. Our previous experiment found that the *AQP1* protein is significantly overexpressed in the kidney of *L. yarkandensis* (Zhang et al., 2019a). Immunohistochemical analysis showed that *ATP1A1* protein is in the basolateral membrane of PCT and TL of the renal medulla, and the *SLC4A4* protein was in the basolateral membrane of renal PCT.

*ATP1A1* protein can establish and maintain a transmembrane electrochemical gradient for Na^+^ and K^+^, whereas *ATP1A1* variants may alter cation channel abnormalities and cause the loss of Na-K-ATPase function (Čechová et al., 2016). This may lead to dysfunction of tubular reabsorption (Lin et al., 2021). *FXYD2* is a regulatory subunit and has been shown to inhibit Na-K-ATPase activity (Mayan et al., 2018). The restriction of *FXYD2* in the distal nephron may play an essential role in basolateral Na-K-ATPase and trans-epithelial sodium reabsorption in the principal cells (Floyd et al., 2010). The presence of Na-K-ATPase with high Na^+^ affinity may be conducive to effective reabsorption of Na^+^ in kidney segments with high Na^+^ load. In conclusion, the up-regulated expression of *ATP1A1* and down-regulated expression of *FXYD2* together increase Na-K-ATPase activity, accelerate the exchange rate between Na^+^/K^+^ in proximal tubules, and maintain ion concentration in proximal tubules to maintain cell homeostasis.

Most of the bicarbonate is reabsorbed from the proximal tubules using NHE (mainly *NHE3*) for H^+^ in exchange for sodium (Wang et al., 2001; Li et al., 2019). The secreted H^+^ binds with HCO_3_^−^ in the lumen and is converted into CO_2_ and H_2_O (Purkerson and Schwartz, 2007). *NHE3* was no differentially expressed in *L. yarkandensis* and *O. cuniculus*, and the protein encoded by the *NHE3* gene was normally expressed in both *L. yarkandensis* and *O. cuniculus*. CO_2_ and H_2_O in the lumen enter the proximal tubule cells through *AQP1* and are converted into HCO_3_^−^ and H^+^ by CAⅡ. The protein *NBCe1* encoded by the *SLC4A4* gene regulates Na^+^-HCO_3_^-^ cotransport in the basolateral proximal tubules, an essential step in bicarbonate reabsorption (Kurtz and Zhu, 2013). HCO_3_^−^ and H^+^ enter the blood at a ratio of 3:1 through *NBCe1* to complete the process of reabsorption (Soleimani et al., 1987). Therefore, the up-regulated expression of the *SLC4A4* protein in the kidney of *L. yarkandensis* can accelerate the bicarbonate reclamation process.

In the vasopressin-regulated water reabsorption pathway, RT-qPCR results showed that *AQP2* was up-regulated, the *ADCY3* gene was significantly up-regulated, and the *CREB3* gene was up-regulated to some extent. Western blotting showed that the *ADCY3* protein was highly expressed in the kidneys of *L. yarkandensis*, and our previous experiment found that *AQP2* protein was highly expressed in the kidneys of *L. yarkandensis* (Zhang et al., 2019a). Immunohistochemical analysis showed that the *ADCY3* protein is highly expressed in CCD and MCD. These results suggest that AVP-mediated water reabsorption mainly occurs in the renal CD. According to the expression of related genes and proteins, the up-regulated expression of *ADCY3* in *L. yarkandensis* can regulate the up-regulated expression of *CREB3* in the nucleus of CD cells, increasing the efficiency of phosphorylated *CREB3* to induce *AQP2* gene transcription and increase *AQP2* protein expression level (Brown, 2003). The up-regulated expression of *ADCY3* may induce the *AQP2* protein to accelerate the transfer from the intracellular storage vesicles to the apical plasma membrane through the *cAMP-PKA-AQP2* level, increasing the number of *AQP2* proteins on the apical plasma membrane, enhanced the water reabsorption capacity of CD cells in the kidney of *L. yarkandensis*. Based on the above studies, we speculated that *L. yarkandensis* could promote water reclamation and maintain water in the body.

### Transcription Factors Involved in Regulating Water Reabsorption

*HIF1A* induces glycolysis by facilitating pyruvate dehydrogenase kinase-1 (*PDK-1*) activity and actively inhibits mitochondrial function and oxygen utilization (He et al., 2014). In the nucleus pulposus, *HIF1A* could also significantly maintain the expression levels of *AQP1* and *AQP5* (Johnson et al., 2015). According to RT-qPCR results, *HIF1A* was up-regulated in the kidney of *L. yarkandensis*, and western blotting analysis showed that *HIF1A* protein was highly expressed. Transcriptomic data were used to predict that *HIF1A* has some regulatory effect on *FXYD2*. Since the down-regulated expression of *FXYD2* and up-regulated expression of *ATP1A1* jointly promote the increase of NA-K-ATPase activity, it is speculated that the up-regulated expression of *HIF1A* can reduce the expression of the *FXYD2* gene–thus indirectly regulating the increase of NA-K-ATPase activity. In the vasopressin-regulated water reabsorption pathway, transcription factor *HIF1A* has some regulatory effects on the *CREB3* gene. Therefore, the up-regulated expression of *HIF1A* can regulate the up-regulated expression of *CREB3*, thereby increasing the transcription and expression of *AQP2* and increasing the water reabsorption capacity of CD cells.

There are four *NFATc* proteins in the *NFATc* subtype transcription factor family, i.e., *NFATc1* (*NFAT2*), *NFATc2* (*NFAT1*), *NFATc3* (*NFAT4*), and *NFATc4* (*NFAT3*) (Hogan et al., 2003). *NFATc* usually collaborates with AP-1 to regulate gene transcription and the transcription of inflammatory cytokines such as interleukin-2 (*IL-2*) (Macián et al., 2001; Reppert et al., 2015). *NFATc3-KO* mice medulla *AQP2* mRNA and protein expression levels are reduced, and integrin-linked kinase (*IKL*) can adjust *NFATc* transcription activity and through the influence of *AQP2 NFATc/AP-1* promoter (Hatem-Vaquero et al., 2017). Other studies have shown that NO can enhance Ca_2_^+^-induced *NFATc* activation, synergistic with Ca_2_^+^, to improve *AQP2* mRNA and protein expression levels in mouse papillae (Albertoni Borghese et al., 2011). Our study found that *NFATc1* was up-regulated in mRNA and protein levels. By contrast, the *CREB3* gene and *AQP2* protein were up-regulated in the kidneys of *L. yarkandensis*, suggesting that the *NFATc1* transcription factor could promote the transcription of *AQP2*.

Studies have shown that *NF-κB* can release P65 at the κB site and increase P50 and P52 monomer binding to inhibit *AQP2* transcription under high osmosis (high tension) (Hasler, 2009). In renal hypertension, decreased AVP sensitivity and increased *NF-κB* activity in renal marrow collecting tubes may lead to decreased *AQP2* expression (Albertoni Borghese et al., 2018). The *NF-κB1* gene and protein expression were up-regulated in the kidneys of *L. yarkandensis*, which seemed to inhibit the expression of the *AQP2* protein. However, another transcription factor, *NFATc1*, was up-regulated, and the expression level was about 2 times higher than the *NF-κB1* gene in protein expression. We hypothesize that the kidney of *L. yarkandensis* requires a high expression of the *AQP2* protein on the CD cell membrane so that *NFATc1* enhances the expression of the *AQP2* protein. The transcription binding site of *NF-κB1* in *AQP2* is located upstream of the transcription binding site of *NFATc1* (Hasler, 2009), is stimulated by high expression of *AQP2*, and leads to the high water content of urine in the urinary lumen; that part of *NF-κB1* gene expression is up-regulated. The inhibition of the expression of the partial *AQP2* protein in the urinary lumen reduces urine volume and reduces the high degree of kidney penetration. In conclusion, We speculate that *L. yarkandensis* can increase the water reclamation efficiency—may be a key adaptation to the arid desert environment.

### Transcription Factor Regulation Model of Kidney Water Reabsorption

**FIGURE 9:**
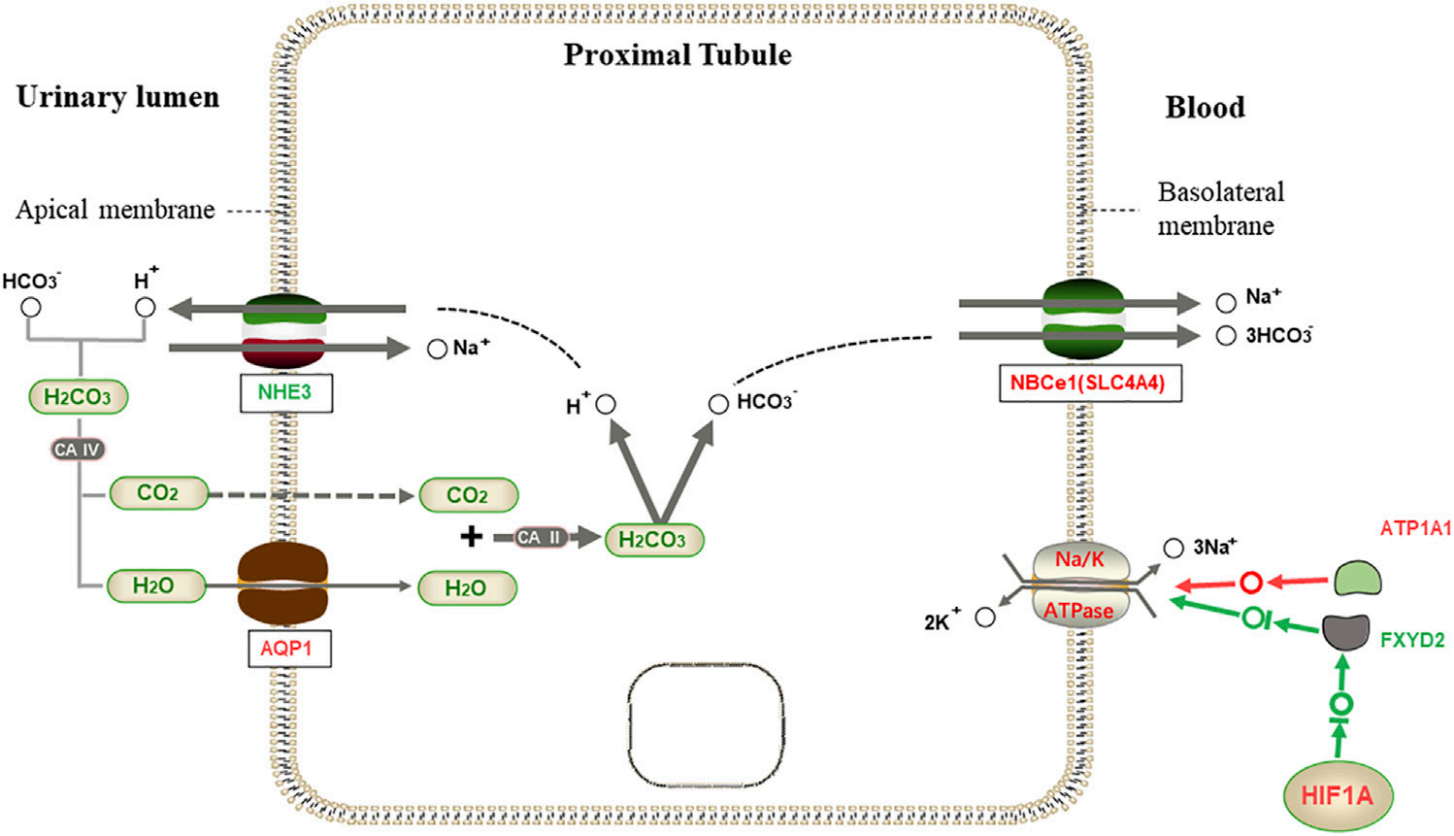
Proximal tubule bicarbonate reclamation. When HCO_3_^−^ is needed by the body, HCO_3_^−^ in the allantoic cavity syntheses H_2_CO_3_ with H^+^ released by Na^+^/H^+^ exchanger (*NHE3*), which is decomposed into CO_2_ and H_2_O under the action of CAⅣ. H_2_O enters proximal tubule cells through aquaporin 1 (*AQP1*), and is synthesized with CO_2_ to produce H_2_CO_3_ under the action of CAⅡ, then decomposed into H^+^ and HCO_3_^−^. Then, Na^+^/3HCO_3_^−^ cotransport is mediated by Na^+^/3HCO_3_^−^ Cotransporter *NBCe1*(*SLC4A4*). However, NA-K-ATPase is transported out of the cell with three Na^+^ on the basolateral membrane, and two K^+^ are exchanged into the cell to maintain the osmotic gradient. Among genes and proteins, red represents up-regulated expression in *L. yarkandensis*, and the green represents down-regulated expression in *L. yarkandensis*. The red arrow represents promoting expression regulation, and the green arrow represents inhibiting expression regulation. Na-K-ATPase α1 subunit (*ATP1A1*) and regulatory FXYD subunit (*FXYD2*) are component subunits of NA-K-ATPase, and *HIF1A* is a transcription factor protein.

We speculate that the regulation of transcription factors and water reabsorption by *L. yarkandensis* kidneys is based on previous studies. When the body is exposed to drought and water shortage, the transcription factor *HIF1A* may be activated to inhibit the expression of *FXYD2*. At the same time, *ATP1A1* is highly expressed, thus jointly enhancing the expression of NA-K-ATPase in proximal tubule cells. The up-regulated expression of the *AQP1* protein and *NBCe1* protein encoded by the *SLC4A4* gene improves the reabsorption capacity of proximal tubule cells to water (Figure 9). This increases the water utilization rate in *L. yarkandensis*.

**FIGURE 10:**
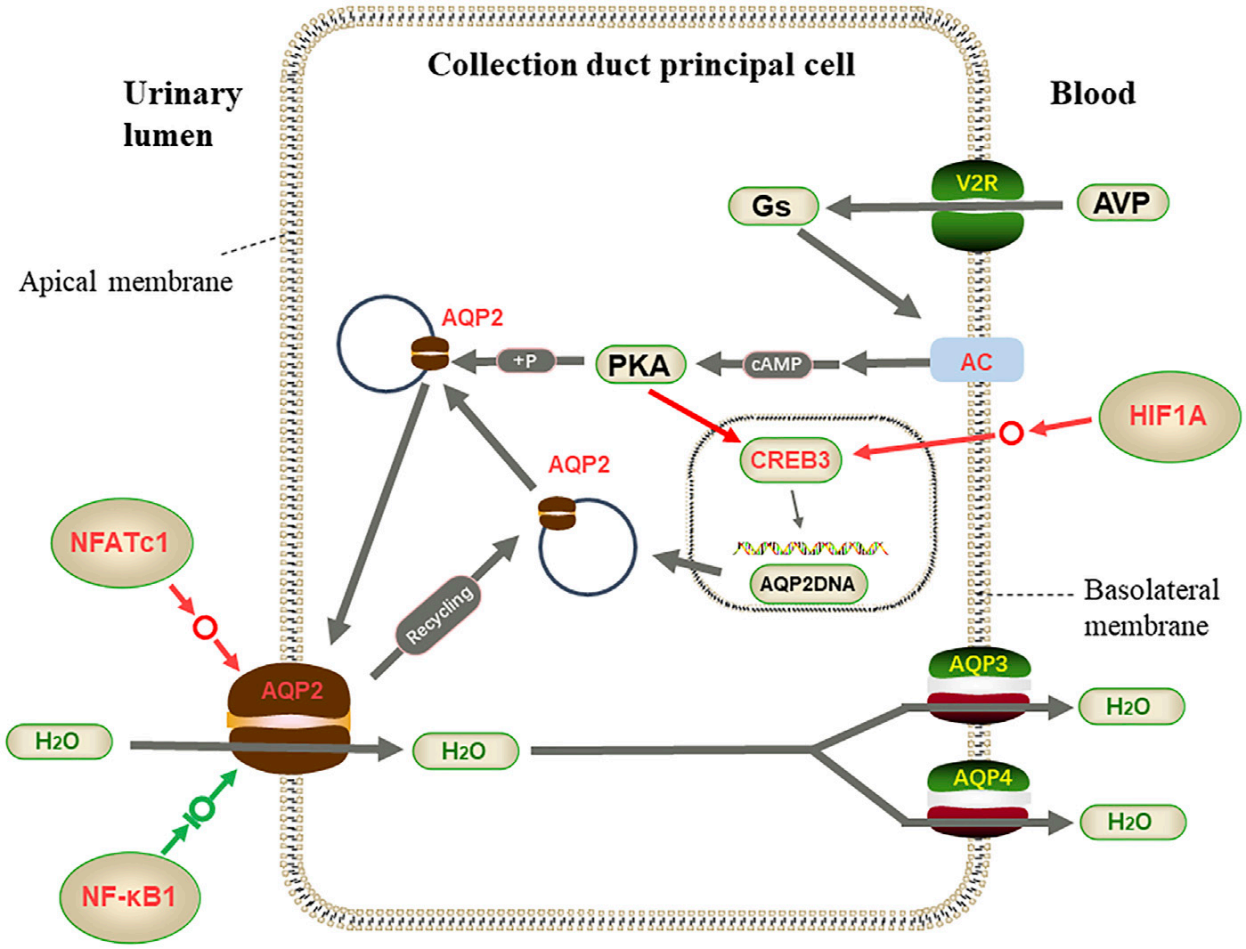
Part of vasopressin-regulated water reabsorption. When the collecting duct is overhydrated or the body is dehydrated, AVP released from the pituitary binds to v arginine vasopressin receptor 2 (AVPR2) to activate adenylate cyclase (AC) and catalyze cyclic adenosine monophosphate (*cAMP*) and Protein kinase A (PKA). Activated PKA phosphorylates aquaporin 2 (*AQP2*) and transfers *AQP2* to the apical plasma membrane, transferring water from allantoic lumen to collecting duct cells by *AQP2* and to blood vessels by *AQP3* and *AQP4*. At the same time, the activated catalytic subunit of PKA enters the nucleus. It phosphorylates *cAMP*-response element-binding protein 3(*CREB3*), which binds to the upstream transcription start site of *AQP2* and activates the transcription of *AQP2*. Among genes and proteins, red represents up-regulated expression in *L. yarkandensis*, and the green represents down-regulated expression in *L. yarkandensis*. The red arrow represents promoting expression regulation, and the green arrow represents inhibiting expression regulation. Hypoxia-inducible factor-1/α(*HIF1A*), nuclear factor of activated T-cell cytoplasmic 1(*NFATc1*), nuclear factor-kappa (*NF-κB*) is a transcription factor protein.

On the other hand, the activated transcription factor *HIF1A* promotes the expression of the *CREB3* gene in the CD nucleus, and the highly expressed transcription factor *NFATc1* also increases the transcription of the *AQP2* gene, leading to the high expression of *ADCY3* as regulated by *AVP*, and increases the *cAMP* level. These changes increase the synthesis of *AQP2* protein in different directions and are likely to accelerate the transfer of *AQP2* protein to the apical plasma membrane. Only the *NF-κB1* transcription factor limited *AQP2* protein synthesis, and together with the transcription factor *NFATc1*, *AQP2* protein expression was in a dynamically balanced range (Figure 10). These adaptations enhance efficient water utilization, helping *L. yarkandensis* cope with the Tarim Basin’s arid desert environment.

## Conclusion

In conclusion, transcriptomic data analysis and pathways related to renal water reabsorption showed that transcription factors *HIF1A*, *NFATc1,* and *NF-κB1* were involved in the renal water reabsorption process of *L. yarkandensis*. It maybe can directly or indirectly regulate the expression of *AQP1*, *AQP2*, *ADCY3*, *ATPA1*, *CREB3*, *SLC4A4*, *FXYD2,* and *SLC9A3*(*NHE3*) genes. Based on the current experimental data, we speculated the partial model of the transcription factor regulating the kidney water reabsorption gene compared with the *O. cuniculus* kidney DEGs. It provides the research foundation for further study on the water balance ability of *L. yarkandensis* kidneys.

## Data Availability

The datasets presented in this study can be found in online repositories. The names of the repository/repositories and accession number(s) can be found below: NCBI BioProject, accession number PRJNA785096

## Supplementary Material

Supplementary Figure S1 Sequence length distribution of the unigene sequences. The X-axis shows the range of lengths of the transcript sequences. The Y-axis shows the number of unigenes.

Supplementary Figure S2 Full western blotting image of some proteins. *ADCY3* and the corresponding *GAPDH* blotting images **(A)**; *NFATc1* and the corresponding *GAPDH* blotting images **(B)**; *NF-kB1*and the corresponding *GAPDH* blotting images **(C)**; *HIF1A* and the corresponding *GAPDH* blotting images **(D)**; *SLC4A4* and the corresponding *GAPDH* blotting images **(E)**; *ATP1A1* and the corresponding GAPDH blotting images **(F)**.
